# Augmented reality board game with multidimensional scaffolding mechanism: A potential new trend for effective organizational strategic planning training

**DOI:** 10.3389/fpsyg.2022.932328

**Published:** 2022-09-12

**Authors:** Huei-Tse Hou

**Affiliations:** Graduate Institute of Applied Science and Technology, National Taiwan University of Science and Technology, Taipei City, Taiwan

**Keywords:** scaffolding, strategic planning, game-based learning (GBL), board game, augmented reality, situated learning, formative assessment

## Introduction

The global trend in the field of employee training and educational innovation emphasizes the training of key competencies (Halász and Michel, 2011; Tiana et al., 2011; Valle and Manso, 2013). The new training trend emphasizes training programs that develop key competencies for organizational members and use these competencies to appropriately solve complex situational problems. Among which, strategic planning is an important ability for governments, businesses, and organizations facing dynamic situations (Bryson et al., 2010; Poister, 2010). Especially in the current complex international environment, with rapid changes in politics, epidemics, wars, economies, and natural disasters, it is crucial to cultivate collaborative and cross-disciplinary talents with sensitivity. Strategic planning can be applied to all areas of strategy development practice.

In the teaching of strategic planning, the provision of contextualized examples is particularly crucial because of the need to analyze specific case contexts. Situated learning (Brown et al., 1989) emphasizes that learners learn in real or simulated contexts, using real work as the main axis of learning, and using context, clues, scaffolding, and diagnostics to promote the depth of learning (Zydneya et al., 2012; Hou and Keng, 2021). If the contextual context and strategic planning tasks are provided and the external environment and internal constraints of the organization are simulated, it is expected that learners can develop the ability to analyze and organize information to make strategic planning and further achieve learning transfer. However, the design of situated learning requires clear goals, feedback, and interaction as well as the ability to motivate learners and provide appropriate support tools to achieve better learning outcomes (Norman, 2014). To promote students' motivation and cognitive engagement in contextual learning, the use of game-based learning is a promising approach. Currently, studies on game-based learning have found positive benefits of games for teaching and learning (Annetta et al., 2009; Hou and Li, 2014; Hou, 2015; Hou and Keng, 2021).

Business simulation games emphasize that players evaluate the benefits, costs, and outcomes of their decisions and learn from analyzing the contextual information of the game (Doyle and Brown, 2000). To simulate real face-to-face interactions in organizations, board games that emphasize physical interactions are suitable for teaching organizational strategic planning. The game mechanics of board games often emphasize interpersonal interaction and mutual collaboration (LeBlanc and Bearison, 2004), and many board games emphasize that players must think strategically to win the game. Several scholars have conducted research on educational board games and found that board games are a positive aid to teaching and learning (von Wangenheim et al., 2012; Hou and Keng, 2021). However, in the context of strategy planning with board games, players often need to analyze more information about the changing context of the external context of the organization. In this case, the use of augmented reality (AR) should help to provide a combination of physical cards and online information.

The strategy-planning game should not only provide participants with a contextual experience but also focus on reflection after the experience. The reflective and adaptive behavior of the participants in the game is crucial, as it can stimulate new strategy ideas (Kiili, 2007) or lead to reflective thinking and behavior (Hou, 2015). A multidimensional scaffold can help learners become more contextualized or engage in cognitive thinking (e.g., Hou et al., 2022). Scaffolds include various types, including cognitive concept scaffolds (guidance on knowledge content concepts), meta-cognitive scaffolds (guidance on how to think), procedural scaffolds (guidance on how to use resources or tools), and strategic scaffolds (guidance on problem-solving strategies) (Hannafin et al., 1999).

In strategy planning, the provision of both meta-cognitive scaffolding and strategy scaffolding is critical. These two types of scaffolds focus on providing learners with guidance on self-planning, monitoring, and evaluation of in-game strategies or lead to thinking about additional problem-solving strategies. In this regard, a board game alone would not be able to provide timely dynamic simulations of a large amount of business data and graphs that follow the implementation of in-game strategy planning and provide learners with immediate meta-cognitive guidance based on this data (e.g., guidance on how to adjust their thinking about strategies). In addition, these scaffolds are useful for diagnosing the learning experiences as formative assessments, which provide feedback and enhance learning performance, and for improving learning motivation and effectiveness. The use of AR technology to provide these scaffolds and diagnostic assessments can provide additional features to stimulate reflection and strategic thinking.

Currently, there is a lack of board games dedicated to strategic planning ability development and a lack of strategic planning games that provide multiple dimensions of scaffolding at the same time. Therefore, this study proposes to use AR technology, situated learning, and multi-dimensional scaffolding theory to plan a multi-scaffolding-oriented AR educational board game framework for strategic planning ability training.

## A multidimensional scaffold-oriented AR board game framework

This study proposes a multidimensional scaffold-oriented AR educational board game framework for strategy planning ability training. This framework integrates multi-dimensional scaffolding (including cognitive scaffolding, metacognitive scaffolding, strategic scaffolding, peer scaffolding, and procedural scaffolding), AR technology, and board game interaction mechanism to provide an integrated game-based interactive environment for players to learn strategic planning in the game.

**Figure 1:**
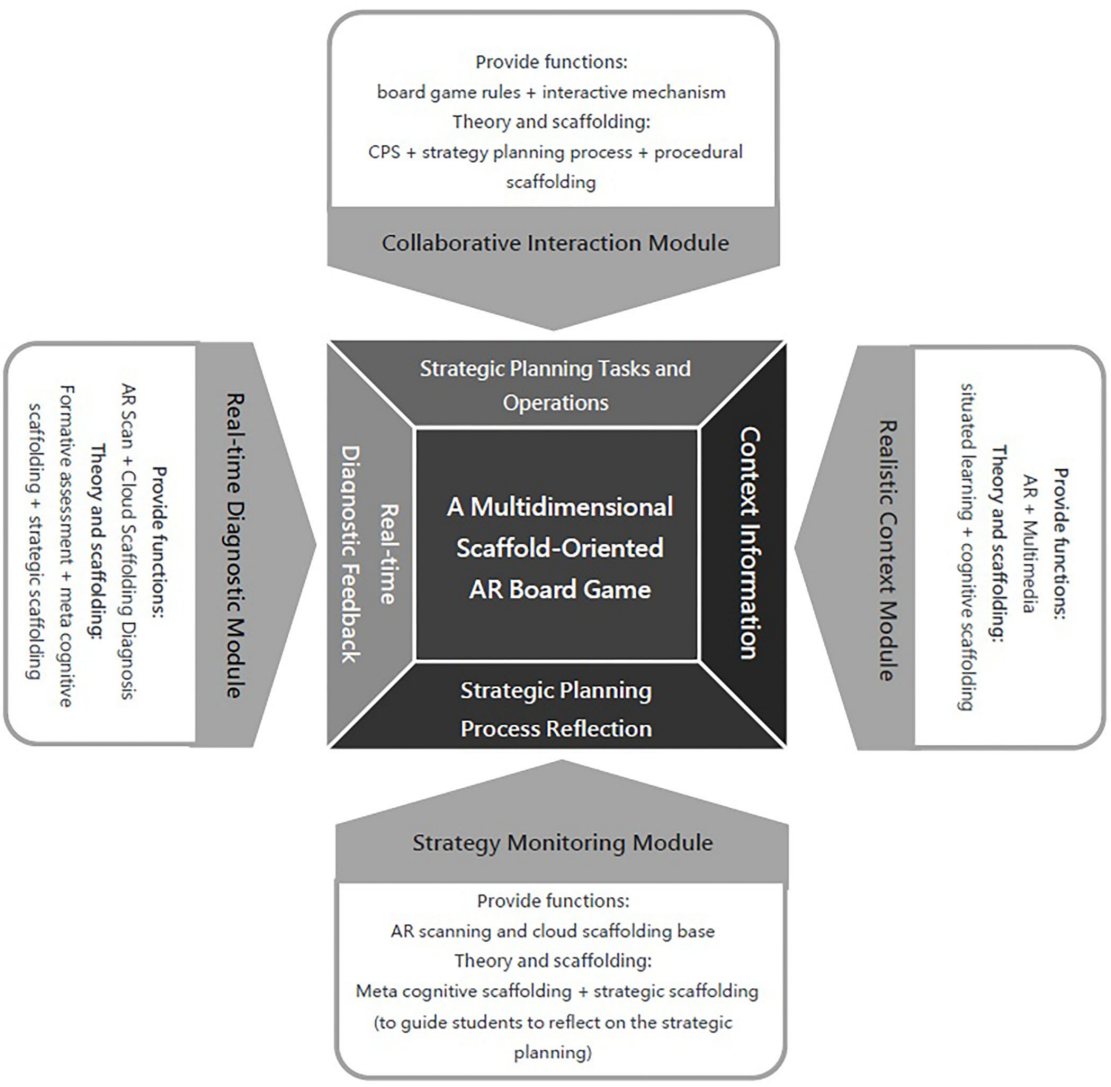
Multidimensional scaffold-oriented AR educational board game framework for strategy planning ability training.

As shown in the Figure 1 below, the entire framework is divided into four modules, which provide four functions: realistic situations, strategy monitoring, real-time diagnosis, and collaborative interaction. Among them, the *realistic context module* will apply the design principles of situated learning (Brown et al., 1989), provide a realistic contextual context through multimedia and AR technology, and provide a cognitive scaffold (Hannafin et al., 1999; Saye and Brush, 2002; Hou and Keng, 2021) to provide a variety of external and internal contextual contexts and scenarios (e.g., news, environmental economic events, competitor news, internal organizational temporal events, or data models) for managing the context as a contextual context for tasks and clues. These clues can be used as cognitive scaffolds to facilitate learning and thinking. In addition, a physical board game is used as a *collaborative interaction module* to design collaborative interaction game rules based on the collaborative problem solving theory (CPS) (Nelson, 1999) to guide groups of learners in the same camp to collaborative problem solving, in which the game card content, game rules, and goal tasks need to be integrated into each stage of the strategy planning process (Hill et al., 2016) to facilitate the alignment of game goals with learning objectives. Players need to conduct resource planning or process planning within the organization to achieve the goals of the strategic planning task. The guide to the strategic planning process can be used as a procedural scaffold to assist players in the complete process of strategic planning.

The *strategy monitoring module* provides a meta-cognitive scaffold that allows learners to scan or enter decision data on specific cards using AR. The module can provide corresponding cues before, during, and after the execution of a strategy based on the data. Finally, the *real-time diagnostic module* provides a formative assessment of the diagnostic and feedback after reviewing the implementation of the player's proposed strategy. Through the scanning of specific combinations of different board cards and the cloud-based scaffolding diagnostics (e.g., diagnostics of strategies and outcomes formed by players by combining related action cards), the app will display various triggered outcomes after the execution of strategies (e.g., diagnostic feedback on the latest value changes and real-time conditions resulting from the implementation of strategy combinations). These formative evaluations can also be used as meta cognitive scaffolds and strategy scaffolds to guide learners to make more in-depth strategy adjustments.

Instructors can use this framework to design board game cards and game interaction rules (e.g., matching, combination, sorting, and other game mechanisms) according to learning objectives. With the common AR picture recognition editing tools on the Internet, various multimedia scaffold designs can be made, and a scaffold-oriented AR educational board game can be completed.

## Discussion

Previous research has found that to promote organizational learning and innovation, there should be a shift to teaching models that promote critical thinking and analytical skills (Dirkx et al., 2006). Previous research has found that strategic planning training lacks experience and feedback in realistic contexts (Ganesh and Sun, 2015), and gaming activities using AR technology in multimedia contexts with multidimensional scaffolding (e.g., Hou et al., 2022) are expected to help address this issue. There is a lack of educational game design framework that combine face-to-face collaborative interactions with multimedia contextual realistic organizational strategy planning activities. The proposed multi-dimensional scaffolding combined with strategy planning theory, situated learning theory, and augmented reality game guidance model should be theoretically innovative and important.

## Author contributions

H-TH: conceptualization, investigation, and writing—original draft.

## Funding

This research was supported by the projects from the US Air Force Office of Scientific Research (AFOSR) project (20IOA038) and the Ministry of Science and Technology, under contract numbers MOST-108-2511-H-011-003-MY3 and MOST-110-2511-H-011-004-MY3.

## Conflict of interest

The author declares that the research was conducted in the absence of any commercial or financial relationships that could be construed as a potential conflict of interest.

## Publisher's note

All claims expressed in this article are solely those of the authors and do not necessarily represent those of their affiliated organizations, or those of the publisher, the editors and the reviewers. Any product that may be evaluated in this article, or claim that may be made by its manufacturer, is not guaranteed or endorsed by the publisher.
